# Collaborative research and knowledge translation on road crashes in Burkina Faso: the police perspective 18 months on

**DOI:** 10.1186/s12961-020-00654-1

**Published:** 2021-01-06

**Authors:** Christian Dagenais, Michelle Proulx, Esther Mc Sween-Cadieux, Aude Nikiema, Emmanuel Bonnet, Valéry Ridde, Paul-André Somé

**Affiliations:** 1 Department of Psychology, Centre-Ville Station, University of Montreal, P.O. Box 6128, Montreal, QC H3C 3J7 Canada; 2 Humanov·Is, 555 René-Lévesque Blvd. W., Montreal, QC H2Z 1B1 Canada; 3 Institut Des Sciences de Societé (INSS/CNRST), Ouagadougou, Burkina Faso; 4 French National Research Institute for Sustainable Development (IRD), UMI Résiliences, Centre IRD de Bondy Nord, Bondy, France; 5 French National Research Institute for Sustainable Development (IRD), CEPED (IRD-Université Paris Descartes), Universités Paris Sorbonne Cités, ERL INSERM SAGESUD, Paris, France; 6 School of Public Health, University of Montreal, Quebec, Canada; 7 Action-Gouvernance-Intégration-Renforcement, Groupe de Travail en Santé et Développement (AGIR/SD), Ouagadougou, Burkina Faso

**Keywords:** Knowledge translation, Research use, Research impact, Collaborative research, Deliberative workshop, Road safety, Public health, Burkina Faso, West Africa

## Abstract

In this commentary, we present a follow-up of two articles published in 2017 and 2018 about road traffic crashes, which is an important public health issue in Africa and Burkina Faso. The first article reported on a research project, conducted in partnership with local actors involved in road safety, carried out in Ouagadougou in 2015. Its aim was to test the effectiveness, acceptability, and capacity of a surveillance system to assess the number of road traffic crashes and their consequences on the health of crash victims. Several knowledge translation activities were carried out to maximize its impact and were reported in the 2018 article published in HRPS: monthly reports presenting the research data, large-format printed maps distributed to the city’s police stations, and a deliberative workshop held at the end of the research project. The present commentary presents our efforts to deepen our understanding of the impacts of the knowledge translation strategy, based on follow-up interviews, 18 months after the workshop, with the heads of the road traffic crash units in Ouagadougou police stations (*n* = 5). Several benefits were reported by respondents. Their involvement in the process prompted them to broaden their knowledge of other ways of dealing with the issue of road crashes. This led them, sometimes with their colleagues, to intervene differently: more rapid response at collision sites, increased surveillance of dangerous intersections, user awareness-raising on the importance of the highway code, etc. However, sustaining these actions over the longer term has proven difficult. Several lessons were derived from this experience, regarding the importance of producing useful and locally applicable research data, of ensuring the acceptability of the technologies used for data collection, of using collaborative approaches in research and knowledge translation, of ensuring the visibility of actions undertaken by actors in the field, and of involving decision-makers in the research process to maximize its impacts.

## Background

The number of road crashes is steadily increasing in Africa and in Burkina Faso, and more specifically in the country’s capital, Ouagadougou [1]. Despite the recent implementation of multiple actions at the national level, road safety is a daunting public health issue [2]. Without access to rigorous and comprehensive data on crashes, injuries, and fatality risks, the ability to implement appropriate and adequate measures is limited [3].

## The TRAUMA research project on road crashes

To alleviate this situation, which is detrimental to population health, a research project on road traffic injuries was carried out in Ouagadougou in partnership with local stakeholders in 2015. Research data from the TRAUMA project have been published elsewhere [2, 4, 6, 7]. Its main results are summarized in the following paragraphs.

The aim of the TRAUMA research project was to test the effectiveness, acceptability, and capacity of a surveillance system to assess the number of road crashes and their consequences on the health of the injured. It was based on a collaborative approach with all the police stations in Ouagadougou (which are responsible for reporting road crashes in the city) and on the use of a surveillance system that was innovative for that setting.

Police officers from each road traffic crash unit of the six police stations and from the central station of the Ouagadougou National Police were involved in data collection. They used geo-tracers (GPS beacons with a telephone chip) to transmit the position of each road crash to a mapping server [4]. At the same time, they completed a crash report form on the circumstances of the collision and the conditions of the people involved. The injured were followed up by the research team in hospital, on admission to the emergency room, and then seven and 30 days after the crash, in line with international standards adopted by WHO [5], to explore the modalities of the care pathway and its implications. Data were collected over a 6-month period [2].

**Fig. 1.**
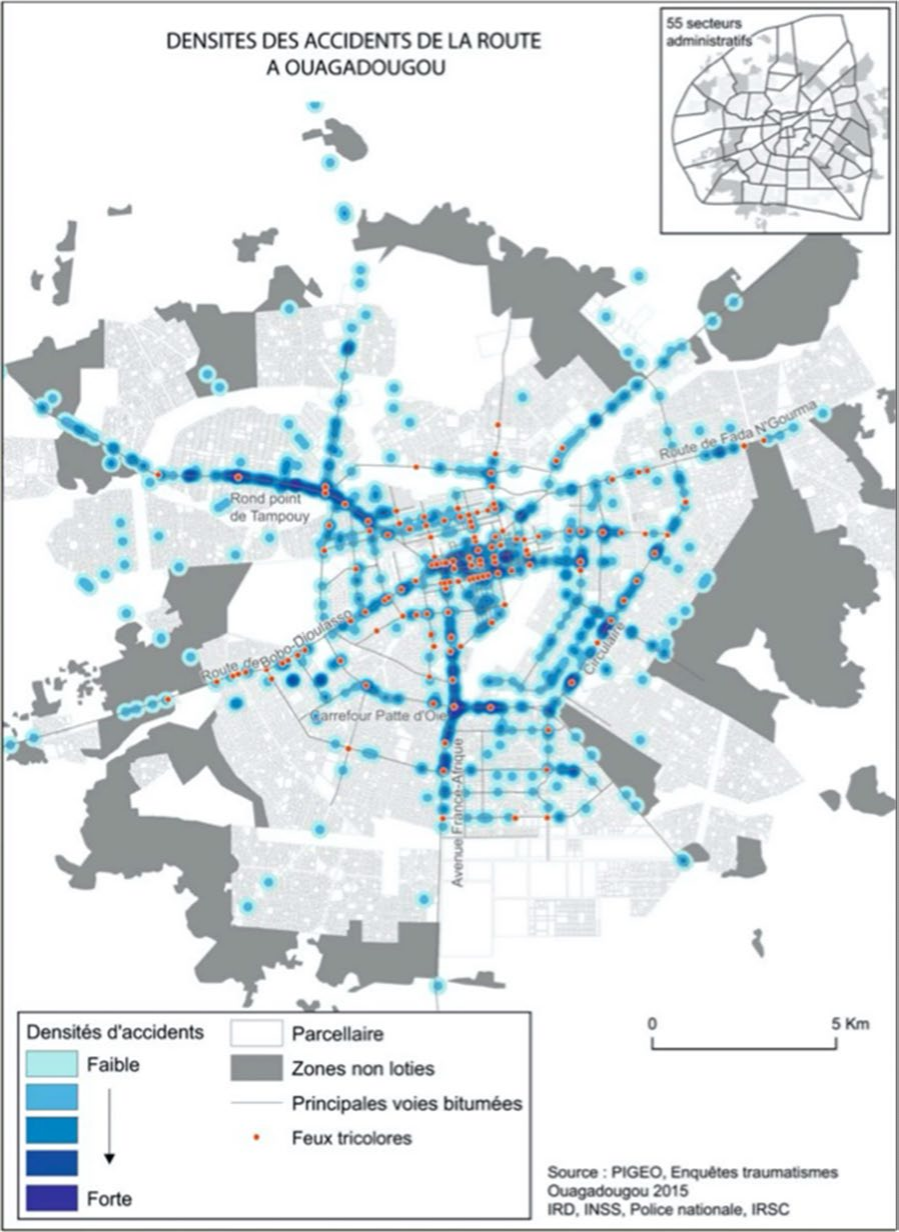
Map of road crashes in the city of Ouagadougou

With this project, it was thus possible to count road crashes, observe their concentration through their geo-locations in Ouagadougou (Fig. 1), and provide estimates of mortality, morbidity, and disability resulting from those crashes. Social autopsies were conducted to better understand both the context of care for the injured and the social representations of road crashes. Another arm of the project examined road safety legislation in Burkina Faso.

## Knowledge translation strategy

Using research evidence to inform practice, decision-making, and public policy is increasingly seen as a core concern, especially in global health [8, 9]. Such use is optimized when sufficiently compelling knowledge translation (KT) strategies are implemented that are based on the sharing, exchange, and transmission of knowledge between researchers and users, thereby combining scientific and experiential knowledge [10]. Accordingly, several knowledge translation activities were undertaken to maximize the impact of the TRAUMA research project and ensure the sharing of research data [11]. The results of the activities carried out by the seven police stations in the capital were provided to the crash unit heads throughout the data collection process in the form of monthly reports. Large-format printed maps presenting the data collected by and for all units were provided for display in the offices of the crash units of each police station.

In addition, a deliberative workshop was held in Ouagadougou at the end of the research project. Organized by the research team, this one-day workshop was aimed at establishing a dialogue between stakeholders and informing policy decisions on road crashes [14]. A deliberative workshop is a knowledge translation strategy that “allow[s] research evidence to be considered together with the views, experiences and tacit knowledge of those who will be involved in, or affected by, future decisions about a high-priority issue” (p. 2) [12]. The workshop brought together local actors working in road safety, including representatives of the police, firefighters, representatives of various non-governmental organizations (NGOs) and civil society associations (CSAs) involved in road safety, as well as representatives of government structures (ONASER—the national road safety bureau), of research centers, of the Ministry of Higher Education, Scientific Research and Innovation, and of the Ministry of National Education, Literacy and the Promotion of National Languages of Burkina Faso. Research briefs were sent to participants a week before the workshop to anchor the discussions in the research data [13, 14]. At this workshop, after reporting the main findings of the study, the research team proposed operational recommendations to improve road safety. Then the various actors in attendance deliberated on the actions to be prioritized and on the means of implementing them.

An evaluation was conducted two to three months after the deliberative workshop to better understand its utility and to measure the potential short-term impacts of the knowledge translation strategy and the research process as a whole. The results of that evaluation were published in HRPS in 2018 [11]. The evaluation showed many positive impacts of the workshop, despite several challenges, including the limited number of high-level policymakers present at the workshop and the difficulty in setting up a committee to follow up on the recommendations after the workshop. According to the participants, the activity fostered a better understanding of the road crashes issue, generated multi-stakeholder dialogue and bilateral learning, and supported professional collaboration, among other positive impacts.

## Methods

To deepen our understanding of the impacts of the knowledge translation strategy over the longer term, and specifically in terms of actions taken by police officers, follow-up interviews were conducted with the heads of the crash units in Ouagadougou police stations. The objective of this follow-up was to document the impacts of the TRAUMA project and knowledge translation activities, including the deliberative workshop, 18 months after it was held. The impacts reported are those perceived, not by the researchers, but by the project partners as represented by the police authorities of the capital.

Three heads of crash units (still in the same position at the time of the interviews) and two retired heads of crash units from five of the seven Ouagadougou police stations agreed to share their views. Unit heads are in charge of the management of crashes in the police station in their territory, with the different police stations being, in turn, overseen by a central police station in Ouagadougou whose mandate is to centralize the city’s crashes data. Respondents were asked about the utility of the research data, the changes observed since the project, mainly in relation to the workshop, and the extent to which these changes were sustained over time.

The interviews were recorded and then transcribed in full. Deductive thematic content analyses (operationalized on the basis of predetermined categories) and inductive analyses (operationalized on the basis of emerging categories) were carried out by a research professional. In this process, the interview transcripts were subjected to repeated readings, then were coded. A lexicon of codes was developed, organized around 13 categories related to knowledge use. The material collected was classified manually into each category and synthesized [15]. Attention was paid to recurrences and divergences in the comments made by the police officers. The summaries were validated by each member of the team who participated in the project. The text was refined as needed by the research professional after further reading of the transcripts. Written consent was obtained from all respondents.

## Results

### Utility and use of research data by the police

The police representatives were in agreement on the relevance and utility of the research data shared at the workshop. Some especially appreciated the fact that the data provided a global view of what was happening in Ouagadougou, especially with regard to the most crash-prone areas of the city. While they were able intuitively to identify road risks in their own units, this data led them to view road risk differently. According to some respondents, the research results made it possible to quantify the risk—that is, to know the probabilities of road crash occurrences, such as their higher incidence at certain intersections and on weekends, as well as the severity of crashes for all units. These results also provided information on the groups most at risk of crashes, mainly involving bicycles and two-wheeled motorized vehicles, notably women and adolescents aged 13 to 15 years.^1^ Only 6% of those injured were pedestrians.

“It’s an outcome of the workshop because it allowed us to tell the authorities which were the intersections where crashes are frequent, and the times at which collisions are reported. If we hadn’t been able to identify that these areas were crash-prone, we wouldn’t have been able to improve safety.”

**Fig. 2.**
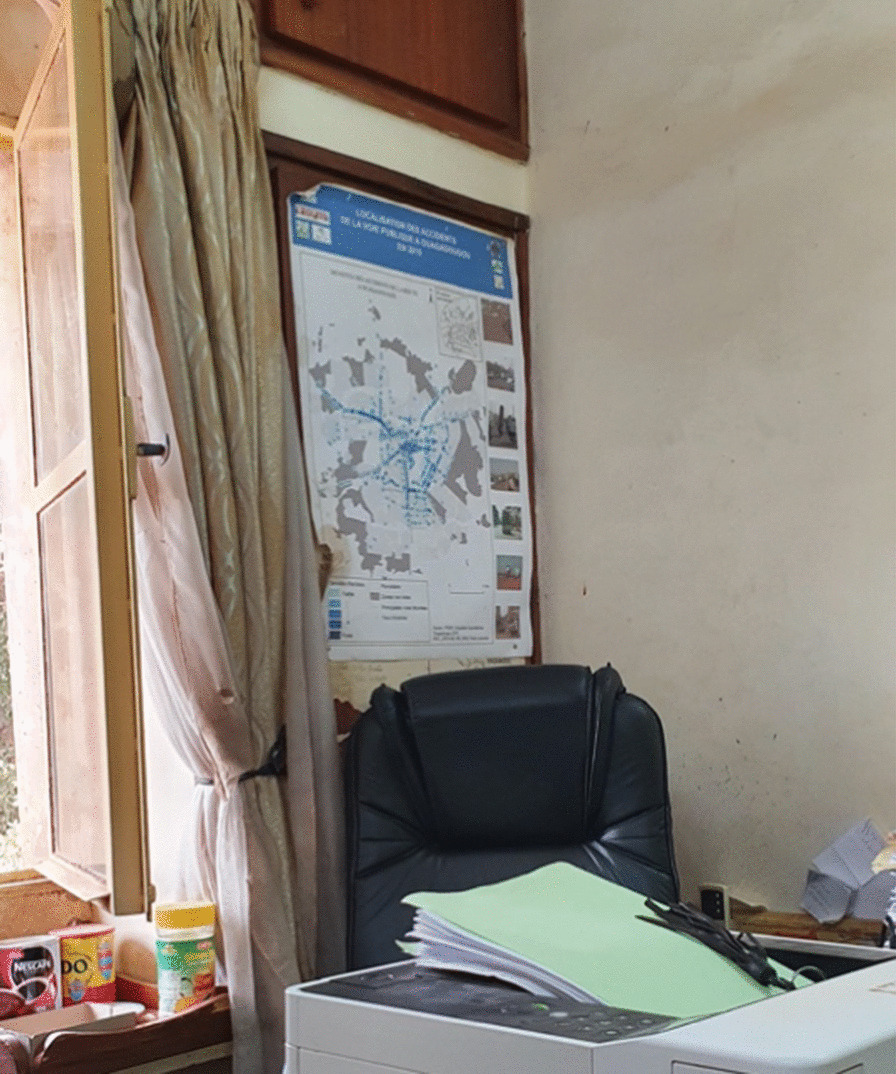
Map on display in a police station in Ouagadougou (December 2019)

The research data, mainly those dealing with the most crash-prone areas, had been put to use. Thus, shortly after the workshop, two unit heads met with their teams to discuss the research data and to identify what was already being done and what should be improved in their unit. One of them studied, with his colleagues, the map provided by the research team describing the crash-prone zones for their unit and for all the units in Ouagadougou. He took the initiative of displaying it in a visible location to raise awareness. In December 2019, the map showing crash concentration zones in Ouagadougou was still posted in the secretariat of the Central Station of the National Police (Fig. 2). According to two respondents, many student police officers and their trainers also used the research data to prepare their theses, based on the tables and other documents provided by the team (e.g. summary sheets of findings).

### New ways of seeing and intervening within teams and sometimes between units

While the research data appeared to be useful, respondents had difficulty commenting on the level of change produced among unit colleagues. Some reported resistance to change by police officers or even the administration. For example, one respondent indicated that it was difficult to influence colleagues and have an impact on the entire territory. Nevertheless, changes were sometimes noted. Some unit heads were troubled by the data on the often difficult experiences of the injured, as these data highlighted significant deficiencies in hospital care. While these data were more relevant to health actors, one unit head indicated that his team now took into account the course and duration of care for the injured in planning face-to-face meetings of witnesses. It is in this step, which precedes the closing of the crash file, that responsibility for the crash is established.

“…our only job was to fill out the reports. We didn’t follow up to see what happened to the injured, except when they come back to our level for the witness meetings…. With the studies we got an idea of the care, how long it can take, how people deal with it…. Now we take into account the duration of treatment before closing the file.”

Another respondent indicated that his team tended to respond more quickly to road crashes, ensuring a timely presence at the scene to clear it and thereby avoid causing further crashes.

“After the workshop, as soon as I got back, I gathered my things together [at the police station], I posted the chart you gave me to my unit so we could study it together… As soon as there’s a crash, we try to react quickly.”

The head of another unit also reported on awareness-raising activities conducted with young people, who often have no knowledge of the highway code. The members of his team took the initiative to download the code onto electronic tablets and make it accessible to crash victims, offering them the possibility of transferring it onto their mobile phones. They talked with the crash victims and discussed road safety with them.

Efforts were also made to raise awareness and enforce sanctions at intersections (e.g. payment of fines, confiscation of vehicles),^2^ according to one unit head.

“We also had to act by imposing fines. Concretely, what had to be done was, not only were you going to pay the fine, but you wouldn’t get your vehicle back for at least two weeks. So it had an impact, it had a positive effect on crashes.”

Finally, one respondent indicated that he now tended to contact his colleagues in other units for assistance in covering a crash site when he was unable to send personnel to the scene.

### Authorities alerted and efforts to increase safety

Beyond the perceived utility of the research results and the implementation of changes by some people, respondents reported a lack of action at the unit and senior authority levels. They pointed to the perceived lack of interest around the issue of road crashes among senior policymakers. One unit head had been made more aware and was brought to see that they had to be reached, they had to really feel the reality of what was happening. Another felt powerless and believed he was too far removed from the decision-making authorities to really be able to instigate change. Some respondents felt that, if another deliberative workshop were to be held, efforts should be made to engage policymakers more.

Nevertheless, it appeared that actions were taken by the political authorities. According to two respondents, a report was transmitted to the authorities, based on the meeting summaries sent to the immediate superiors in each unit. The policy level did, in fact, receive this report and decided to put it into action, hence the increased security at intersections about 6 months after the workshop. Additional budgets were allocated and an increased police presence was observed, particularly at the most crash-prone intersections with traffic lights, which corroborated the data produced by one of the spatial analyses conducted as part of the project. That analysis showed that the number of crashes was much higher at intersections with traffic lights than at other intersections [16]. The increased security at intersections was implemented at the initiative of the administrative and political authorities, who had been alerted and made aware of the issue.

### Difficulty of sustaining changes

There appeared, however, to have been a slackening of road surveillance over time. One respondent even felt they were back to square one, and others concurred. It was not possible to confirm this information, nor to speculate on the real causes of this apparent backsliding. Some attributed it to a lack of staff (e.g. staff turnover) and of material resources in the units. For example, for almost 6 months, one unit was unable to conduct any outings due to a lack of functioning vehicles. During that time, the central police station took on the task of preparing reports. Others suggested that personnel changes at the administrative level and a lack of resources at the policy level may also have had an impact.

One unit head, on the other hand, believed surveillance had been sustained, but with less rigor. He noted that police presence was now random rather than continuous at the designated intersections. In his opinion, there had been a relaxation of awareness-raising activities and sanctions, such that users were less afraid of the police. That respondent felt, however, that people needed to be made more aware. The current actions were not adequately reaching users, who were often very young and illiterate, did not know how to drive, or drove without a license.

To ensure the sustainability of the changes initiated, some respondents felt it would be necessary to continue alerting the authorities, drawing their attention to traffic crashes and their consequences, and urging them to break the chain of civic irresponsibility among both users and police officers (very few of whom wore helmets in traffic). Action was also needed on currently defective road infrastructures (e.g. lanes often narrow and congested) and on the organization of police work.

### Desire to sustain data collection

Continued data collection would also be highly desirable to better assess the evolution of road crashes in Ouagadougou. Are the areas identified still active? Have the road risks taken other forms or have they shifted elsewhere? Some respondents would have liked to continue receiving information on crash-prone areas, but this was apparently no longer the case once the funded research project ended. According to one respondent, such data are necessary and can be useful when advocating with authorities for additional resources.

Anything that is digital and increases the efficiency and speed of work appeared to be well received by respondents. Several noted that they appreciated the use of geo-tracers. According to one unit head, the tool allowed direct transmission of information, although it sometimes presented operating problems (e.g. geo-tracer malfunction) [4]. The option proposed by the team of using a smartphone, on the other hand, seemed ideal. Respondents acknowledged that the technology allowed both geo-location and note-taking with download capabilities. The technology enabled rapid activation of results and rigorous data collection on an ongoing basis.

## What we have learned

This follow-up offers new and useful lessons about the impacts that a collaborative research project accompanied by knowledge translation activities can have, even despite certain limitations. It would have been interesting to obtain the views of other stakeholders present at the workshop, as well, and to contrast them with those of the five unit heads interviewed, who were generally positive about the project. Nevertheless, according to the respondents, the research and knowledge translation activities produced certain benefits. From this experience, we can identify five lessons learned, based on the data presented in this report.

1. *Produce research data useful to actors in the field.* As mentioned, effects were observed, shortly after the workshop, in terms of increased road security, presumably following a report sent to the authorities. It seemed that there was a willingness, at that time, on the part of the public authorities to correct the situation through increased police presence at intersections, and that awareness-raising activities and sanctions were increased. Identification of the most crash-prone locations appeared to have been essential data that aroused considerable interest and mobilized efforts at the level not only of the units but also, initially, of the public authorities. These results show that research evidence, when it is credible, useful, and adapted to the needs of users, is much more likely to be applied [17, 18].
2. *Ensure the acceptability of the technologies used for data collection.* The use of a geo-tracer appeared to be helpful and appreciated. Respondents spoke positively about its use. The ability to communicate and transmit road crash data quickly was appreciated. On this point, the option offered by the research team to use a smartphone seemed to be welcomed in this project. The team’s expertise also showed that the use of smartphones had also been appreciated in other research projects in Benin and Mali and could help enhance local actors’ sense of empowerment. In Burkina Faso, the technology showed promise for providing, on an ongoing basis, rigorous data on which to base actions to reduce crashes and alert the authorities [4]. It also showed that it was possible to implement new data collection methods or tools, when police officers in the units normally only had access to paper crash report forms, a result that is relevant in many other sectors, such as health. Further research activities, using the same technology, were seen as a means of ensuring the continued availability of rigorous data. However, depending on research funds to pay the telephone charges to transmit data appears to be a major impediment to the sustainable use of the technologies.
3. *Use collaborative approaches to research and knowledge translation.* The collaborative research approach appeared to have facilitated the uptake and use of research evidence. This approach, in which police officers from each unit participated actively in using geo-tracers and filling out report forms, was shown to be effective for collecting data that were sensitive to local contexts and their local applications. The interactive and inclusive nature of the deliberative workshop also seems to have had a positive impact [11]. It is conceivable that in the workshop discussions, with the brainstorming of ideas about the research evidence and its endorsement by peers, participants’ intentions to take action on road crash prevention were reinforced [14, 19].
4. *Give visibility to field actors to provide them with more effective action mechanisms.* The selected technology not only provided geo-localized statistics, it also enhanced the crash unit’s visibility in police stations. In addition to producing data, the geographic approach, by documenting precise locations, which no scientific or administrative work had done before, seemed to confirm the knowledge and the intuitions of the crash unit teams. This empowered them to advocate with their superiors. The approach also appeared to give them visibility. The crash unit is just one of many services within police stations. While it is an obligatory service, since there are no joint crash reports in Burkina Faso, the unit produces data for annual reports that are useful to know, but unfortunately not well structured and little used, or even forgotten. In this research project, the cartographic data made visible the field activities of the seven police stations. It also provided a capital-wide perspective on the actions undertaken with regard to road crashes, rendering the phenomenon more visible and of greater interest to the authorities.
5. *Engage high-level decision-makers more in the process to maximize research impacts.* It appeared that the initial actions carried out by public authorities were not sustained and that there was a relaxation of police surveillance at intersections. It was not possible to determine precisely whether this slackening was due to a lack of political will or of human or material resources—a glaring problem in Burkina Faso—or to other factors, such as the public security issues that arose during this period [4]. However, while efforts were undeniably made at the unit level to alert the public authorities, it was impossible to determine whether there was any real change at the policy level. This reaffirms the importance of ensuring the presence of decision-makers at knowledge translation activities to make effective change possible [14, 20]. This also underscores the importance for researchers of focusing on attracting decision-makers’ interest, convincing them of the added value of their recommendations, and persuading them to support the changes needed to put them into practice [10].

## Conclusion

In conclusion, this experience has shown that implementing a partnership-based research project that includes a knowledge translation process can enable actors in the field and researchers to work collectively to improve road safety. While the research methodology was appreciated and the data produced were perceived as useful in the field, additional efforts will be needed to ensure that longer-term effects are produced and sustained at the policy level.

As of this writing, a new project is underway. It uses the same technology and relates to the same territory. Eighty-seven police officers have just received training to be able to geo-locate accidents in Ouagadougou. The results of this project will be available within 18 months.

## Data Availability

The datasets generated and analyzed during the current study are not publicly available due to confidentiality constraints but are available in French from the corresponding author upon reasonable request.

## Abbreviations

CSA: Civil Society Associations
GPS: Global positioning system
KT: Knowledge translation
NGO: Non-Governmental Organizations
ONASER: The National Road Safety Bureau

## Footnotes

1 The minimum age to obtain a driver’s license for a motorcycle less than 125 cc is 14 years, but many people drive without a license.

2 In the transcripts there is no indication as to the types of traffic violations that led to sanctions.
